# Diagnosis and therapeutic challenges in the exploration of cyberchondria

**DOI:** 10.1192/j.eurpsy.2025.1037

**Published:** 2025-08-26

**Authors:** O. Vasiliu, A. M. Ciobanu

**Affiliations:** 1 Neuroscience Department, “Carol Davila” University of Medicine; 2 Psychiatry Department, “Dr. Carol Davila” University Emergency Central Military Hospital; 3 Psychiatry Department, „Prof. Dr. Alexandru Obregia” Clinical Hospital of Psychiatry, Bucharest, Romania

## Abstract

**Introduction:**

Cyberchondria is usually defined by health-related worries and repeated searches on the Internet for data on health conditions, although this behavior is associated with significant discomfort, distress, or anxiety. However, no consensus on a definition exists, and the nosography of this disorder is still uncertain, as it is not recognized by any of the currently available classifications of mental disorders. Still, cyberchondria has been associated with a negative impact on the quality of life and comorbid anxiety-depressive clinical manifestations (Ambrosini et al. *Heliyon* 2022;8(5) e09437), thus highlighting the need for early detection and treatment of this disorder.

**Objectives:**

This review aimed to assess the data supporting the diagnosis and therapeutic management of cyberchondria and to identify areas for further research in this field.

**Methods:**

The review included three databases (Google Scholar, PubMed, and EMBASE), explored from their inception to June 2024, for papers published in English using the keywords “cyberchondria,” and “diagnosis,” “clinical scales,” or “treatment.”

**Results:**

According to data extracted from 34 primary and secondary sources, health anxiety (HA) was positively correlated with seeking online information about health and with cyberchondria. Studies exploring the overlap between HA and cyberchondria have found the two conditions were distinct on multiple measures, including functional impairment and healthcare resources use. The construct of cyberchondria has several dimensions, such as “compulsion,” “distress,” “excessiveness,” “reassurance,” “mistrust,” “illness-related Internet use,” and “metacognitive beliefs,” which are explored by specific structured methods. Two questionnaires have been created for this purpose, i.e., the Cyberchondria Severity Scale (CSS) (McElroy & Shevlin *J Anxiety Disord* 2014;28(2) 259-65) and the Online Health-related Beliefs and Behaviours Inventory (Singh & Brown *Anx Stress Coping* 2014;27(5) 542-54). Prevention should target Internet users’ expectations (avoiding self-diagnosis, verifying data on the Internet with a health specialist, and searching for low-quality information on unofficial sites). Group cognitive-behavior therapy delivered by the Internet was associated with favorable results, based on the CSS scores, but there is a need for further, larger group studies to confirm these observations.

**Conclusions:**

Cyberchondria still needs extensive explorations to be defined as a nosographic stable condition, although dimensions of this concept have begun to be explored in a systematic manner, and studies investigating psychotherapeutic approaches for this disorder have been initiated. Due to the continually increasing access of the general population to medical data online, the exploration of HA and cyberchondria is expected to attract more interest from mental health specialists in the near future.

**Disclosure of Interest:**

None Declared

